# Different response of perennial ryegrass—*Epichloë* endophyte symbiota to the elevated concentration of heavy metals in soil

**DOI:** 10.1007/s13353-021-00661-0

**Published:** 2021-09-21

**Authors:** Grzegorz Żurek, Barbara Wiewióra, Krystyna Rybka, Kamil Prokopiuk

**Affiliations:** 1 Department of Grasses, Legumes and Energy Plants, Plant Breeding and Acclimatization Institute National Research Institute, Radzików, Poland; 2 Department of Seed Science and Technology, Plant Breeding and Acclimatization Institute National Research Institute, Radzików, Poland; 3 Department of Plant Physiology and Biochemistry, Plant Breeding and Acclimatization Institute National Research Institute, Radzików, Poland

**Keywords:** *Epichloë* endophytes, Heavy metals, Perennial ryegrass, Photosynthesis, Phytoremediation, Soil pollution

## Abstract

The phenomenon of plant mutualistic symbiosis with microbes may have a positive effect on the improvement of plant tolerance to environmental stresses as well as on the ability of plants to accumulate heavy metal (HM) ions from soil. The influence of *Epichloë* fungal endophyte (*Ascomycota*, *Clavicipitaceae*) on perennial ryegrass (*Lolium perenne* L.) plants grown in the presence of elevated concentrations of HM ions (Cd^2+^, Pb^2+^, and Cu^2+^) in soil was studied. The presence of *Epichloë* in the host grass tissues resulted in different accumulation of HM ions in the aboveground parts of the plants. In some cases, endophyte infection positively affected ryegrass ability to accumulate HM ions from soil. In plants with (*E* +) and without (*E* −) endophytes, the hormesis effect was induced by the elevated concentration of Cu^2+^ ions, resulting in better growth and photosynthesis, as examined by measurements of Chl *a* fluorescence. The obtained results indicate that based on the laboratory evaluation of the efficiency of HM accumulation, we were able to choose the best associations of perennial ryegrass with endophytes for HM phytoremediation.

**Supplementary Information:**

The online version contains supplementary material available at 10.1007/s13353-021-00661-0.

## Introduction

Endophytes can colonize plant tissues and live without inducing any visible symptoms of biotic stress in plants. In general, as a consequence of host plant–microbe interactions, these endophytes produce a range of alkaloids and stimulate the host plant for enhanced synthesis of primary and secondary metabolites, e.g., free sugars, sugar alcohols, proline, glutamic acid, phospholipids, proteins, and polysaccharides (Avila et al. 2012; Bush et al. 1997; Nagabhyru et al. 2013; Porter 1994; Rasmussen et al. 2008, Soto-Bajas et al. 2016). Hao et al. (2010) observed that treatment of suspension cells of *Ginkgo biloba* with fungal endophytes resulted in the accumulation of flavonoids, increased abscisic acid (ABA) production, and activation of phenylalanine ammonia-lyase (PAL). Also, the root metabolism is altered in response to colonization of the aboveground parts of plants (Strehmel et al. 2016; Slaughter et al. 2018). Altogether, the mutual associations lead to changes in host plant gene expression and improve plant adaptations to environmental stresses, both biotic (e.g., insects, herbivore animals, diseases) and abiotic (e.g., drought) (Bacon et al. 2015; Dupont et al. 2015; Rodriguez et al. 2008; Schardl et al. 2012, 2013).

Inhibition of photosynthesis by heavy metals (HM) has been well documented (Clijsters and Van Assche 1985; Prasad and Strzałka 1999; Singh et al. 2011). HM stress induces a series of biochemical and physiological modifications in plant tissues that display common characteristics with those induced by drought. Membrane damage and altered enzyme activities lead to a wide range of secondary effects that concern practically all the physiological processes (Barceló and Poschenrieder 1990). Photosynthesis is a very sensitive process due to several structural and metabolic disturbances, like direct interactions of HM ions with thiol, histidyl- and carboxyl- groups of cell proteins, induction of reactive oxygen species (ROS) formation, and displacement of essential cations in protein active centers (Hall 2002; Hossain et al. 2012; Farid et al. 2013). Some ions such as Hg^2+^, Cu^2+^, Cd^2+^, Ni^2+^, or Zn^2+^ may substitute the central Mg^2+^ ion in chlorophyll molecules, forming complexes lowering the quantum efficiency of PSII (Van Assche and Clijsters 1990; Sharma and Dietz 2009). These circumstances affect most of the parameters of chlorophyll *a* (Chl *a*) fluorescence detected by the JIP test (Żurek et al. 2014). However, it has been demonstrated that endophytes play a key role in host plant adaptation to polluted environments and that they can enhance phytoremediation by mobilizing/degrading or immobilizing contaminants in the soil, promoting plant growth, decreasing phytotoxicity and improving plants’ HM ion tolerance (Soleimani et al. 2010; Li et al. 2012a, b; Li et al. 2016).

Species of the fungal genus *Epichloë* (*Ascomycota*, *Clavicipitaceae*) are specialized fungi of cool-season grasses that can grow throughout the aerial parts of their host plants, forming systemic and predominantly asymptomatic associations, resulting in defensive mutualism (Clay 1988; Tadych et al. 2014). The importance of *Epichloë* endophytes for ecosystems due to modulation of both below- and aboveground ecosystem processes is well recognized and accepted (Saikkonen et al. 2016).

Phytoremediation is increasingly used as a sustainable approach for soil remediation. However, methodology improvement is constantly forced due to the expected increase in phytoremediation efficacy as well as due to economic reasons. Due to complex biological interactions, currently used methods do not always give the demanded results, so further multidirectional studies are needed (Thijs et al. 2017).

The aim of this study was to describe the different reaction of perennial ryegrass—*Epichloë* endophyte associations to the elevated concentration of lead, cadmium, and copper in soil with further possible application in the phytoremediation process.

## Materials and methods

### Plant collection

**Fig. 1:**
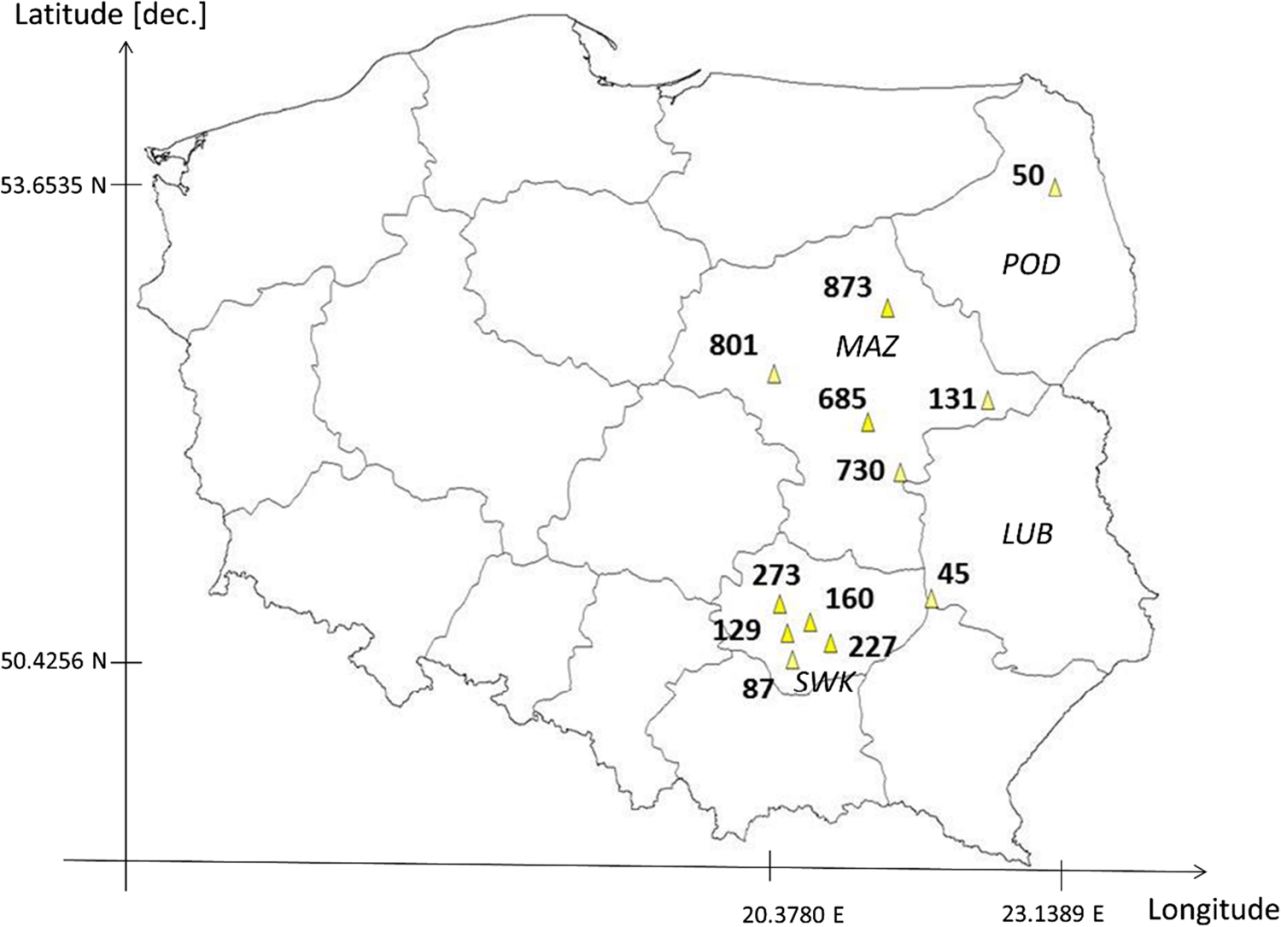
Map of the distribution of ecotype collection sites in regions of Poland: *POD* (Podlaskie), *MA*Z (Mazowieckie), *LUB* (Lubelskie), *SWK* (Świętokrzyskie). Ecotypes are identified by reference numbers the same across the whole manuscript. Map drawn with the DIVA-GIS ver. 7.1.7 software (http:///www.diva-gis.org)

Ecotypes of perennial ryegrass (*Lolium perenne* L.) were collected from 12 localities in Poland in the form of living plants from permanent grasslands in most cases used for cattle feeding. The term “ecotype” refers to a group of plants within a species that is adapted to particular environmental conditions (locality) and therefore exhibiting structural or physiological differences from the other members of the same species. Those areas were located in Podlaskie (POD), Mazowieckie (MAZ), Lubelskie (LUB), and Świętokrzyskie (SWK) regions located on Central European Plain, in Poland. (Fig. 1, Tab. 1, Supp. Tab.1).

**Table 1:** Analysis of variance for the effect of ecotype, HM presence in soil and endophyte on yield of aerial parts of plants and CCI values. *F* values were given and significance of the effects and interactions with probability higher than 99.9% (***) or 95% (**)

Source of variation	Aboveground biomass collected in:				CCI
	1st cut	2nd cut	3rd cut	All cuts (sum)	
Main effects:					
Ecotype [1]	2.75^**^	3.64^***^	0.86^ ns^	1.94^**^	2.94^***^
HM in soil [2]	97.05^***^	867.79^***^	205.01^***^	455.9^***^	98.90^***^
Endophyte presence [3]	4.03^**^	5.86^**^	0.05^ ns^	0.05^ ns^	2.41^ ns^
Interactions					
Ecotype × HM	2.36^**^	1.90^**^	0.91^ ns^	1.72^ ns^	4.05^***^
Ecotype × endophyte pres	0.85^ ns^	1.62^ ns^	1.15^ ns^	1.39^ ns^	0.95^ ns^
HM × endophyte pres	0.30^ ns^	2.60^ ns^	2.06^ ns^	0.78^ ns^	0.01^ ns^
[1] × [2] × [3]	1.23^ ns^	1.62^ ns^	0.81^ ns^	1.34^ ns^	1.78^ ns^

From five to ten plants were picked up in each locality, with the distances of 5–10 m from each other, to avoid collecting clones. GPS coordinates of each locality were recorded. Average concentration of Pb^2+^, Cu^2+^, and Cd^2+^ ions in soils for regions of collection sites were given as per Terelak (2007).

Since plants in the described experiment were part of a large collection, their numbering has no ordinal values. Collected ecotypes were further replanted in a spaced nursery, with 0.5 m distances between plants in Radzików, Poland (52.21 N; 20.64 E). No additional treatments (fertilization, watering, chemical weed control) were applied.

### Endophyte detection

Perennial ryegrass–*Epichloë* endophyte associations were identified by the rapid staining method according to Saha et al. (1988). Fungal hyphae (*E* +), appeared as an intercellular, long, and convoluted hyphae parallel to the leaf-sheath axis of the plant cell without forming haustorial structures (Clay and Holah 1999), (Supp. Fig. S1). On the bases of our recent discoveries and work done on perennial ryegrass endophytes (Wiewióra et al. 2015a, 2015b), fungus forming fungal hyphae inside intercellular spaces were described as belonging to genus *Epichloë* (*Clavicipitaceae*). Based on studies with confirmed isolates describing the typical fungal hyphae in the intercellular space of infected tissues from perennial ryegrass, and our own recent studies, we refer to the endophyte found in Polish perennial ryegrass as *Epichloë festucae* spp. *lolii*.

Twelve ecotypes with *E* + plants were selected as material for further studies. Seeds were collected only from *E* + plants of those ecotypes grown in the nursery and again tested for the presence of the endophyte hyphae using the rose bengal staining method (Saha et al. 1988). Half of the seeds from each ecotype was treated with Tebuconazole (placing the seeds in a liquid suspension), a triazole fungicide to remove the endophyte from seed bulk (*E* −). Both *E* + and *E* − seeds were sown on filter paper and seedlings were transferred to 0.5 l pots filled with mixture (1:2) of sterilized sand and peat. Seedlings were grown in pots for 4 weeks, with frequent watering and without additional fertilization.

The presence/absence of the endophyte hyphae was again confirmed on 3–4 weeks old seedlings by rose bengal staining before microscopic examination of 3 tillers per each plant. For each ecotype, 12 *E* − and 12 *E* + plants were vegetatively propagated: half of each set was intended for HM treatment and half remained as a control (no HM). As a result of the final round of vegetative propagation, 24 plants per ecotype *E* + and the same number per *E* − were used in the experiment run in fourfold repetitions per 3 plants each. Again, the endophyte status (*E* + /*E* −) was checked.

### Pot experiment

From each ecotype for both *E* + and *E* − forms, 24 plants were planted, 3 in one 1.5 L pot containing a mixture (1:2) of sterilized sand and peat substrate of the final content of: 95.1 N; 150.2 P_2_O_5_; 153.3 K_2_O; 55.5 MgO; 7.7 Pb^2+^; 0.2 Cd^2+^; and 2.4 Cu^2+^ [mg·kg^−1^ of dry substrate]; pH = 6.1 and 13% of soil organic carbon (SOC).

The pot experiment was arranged into a randomized complete block design with 4 blocks, where each ecotype was grown in 4 pots per block (3 plants per pot): two pots with *E* + plants and two pots with *E* − plants. From those four pots, two were treated with HM solution (see below) and two were control. Pots in blocks were re-arranged during the experiment to reduce the positional effect and reduce the residual or pot-to-pot variance. Therefore, two factors were used in the aforementioned experiment: the first, endophyte infection (*E* + and *E* − plants) and the second, HM treatment.

The experiment was run in a glasshouse, starting from late spring for 16 weeks in total, with the first 7 weeks of HM treatment. Seedlings were planted into pots, and after 3 weeks of growth in the glasshouse, the first watering was applied; then, watering was applied 9 times during the next 36 days of growth. Control pots were watered with distilled water. Intervals between watering usually were 4–5 days. The whole watering brought in total 20 mg of Cd^2+^ and 700 mg of both Pb^2+^ and Cu^2+^ ions in 1 kg^−1^ of the used substrate. Finally, HM ion concentration in the substrate, as determined by Regional Agrochemical Station in Warsaw (accredited laboratory acc. PN-EN ISO/IEC 17,025:2005), reached: 15.5 Cd^2+^; 550.9 Pb^2+^; 546.0 Cu^2+^ [mg·kg^−1^].

### Analysis of biomass yields, relative chlorophyll contents, and Chl a fluorescence parameters

Biometric phenotyping of the aboveground part of plants was done to determine the rate of plant growth. Three cuts of plants from all experimental pots were done after 1, 2, and 4 months of plant growth in pots since planting, followed by drying at 70 °C for 3 days for determination of dry matter yield. Dry biomass from each pot was collected to determine HM concentration in plants.

Chlorophyll content index (CCI) was measured with CCM200 Plus (PSI, Brno, Czech Republic), on 3 leaves per plant for a total of 24 plants of both forms *E* + and *E* − of each ecotype. The single result consisted of five single measurements per leaf.

Chlorophyll *a* (Chl *a*) fluorescence was measured using PocketPEA portable fluorimeter (Hansatech Instruments, King’s Lynn, Norfolk, UK). Three measurements per plant (3 plants per ecotype per replication per variant) were done. Fluorescence was induced by saturating, red actinic light with energy of 3.500 μmol·m^−2^·s^−1^. Measured and calculated parameters were used for the interpretation of endophyte-plant interaction in the presence of HM ions (Paunov et al. 2018). Measured parameters: *F*_O_ ≈ *F*_50µs_ [minimal fluorescence]; *F*_M_ = *F*_P_ [maximal recorded fluorescence]; *T*_FM_ [time (in ms) to reach the maximal fluorescence, *F*_M_]; Area [total complementary area between the fluorescence induction curve and *F*_M_ of OJIP curve]. Parameters calculated and listed by the PocketPEA software: *F*_V_ [maximal variable fluorescence calculated as *F*_M_ – *F*_O_]; *F*_V_/*F*_M_ [force of the light reactions]; RC/ABS [the amount of active reaction centers per absorption]; (1-*V*_J_)/*V*_J_ [measure of forward electron transport]; PI_ABS_ [performance index]. The above measurements (CCI and Chl *a*) were done 2 weeks after the last HM ions dosing.

### Chemical analysis

Determination of HM concentration in plants and soil were done as described previously (Żurek et al. 2014) by Regional Agrochemical Station in Warsaw (accredited laboratory acc. PN-EN ISO/IEC 17,025:2005). Plant material was washed with tap water and then with deionized water in an ultrasonic washer to remove all soil particles followed by drying at 70 °C for 3 days. Three hundred mg of dried, ground plant material was wet-washed using concentrated nitric acid (Merck) in a microwave system (MDS 2000, CEM, USA).

For determination of total HM ion (Cd^2+^, Pb^2+^, and Cu^2+^) concentration in soil, extraction of air-dried soil samples was taken at the end of the experiment from each pot, ground to < 0.25 mm and extracted with concentrated perchloric (HClO_4_) and fluoric (HF) acids. The amount of Cd^2+^, Pb^2+^, and Cu^2+^ ions were measured using inductively coupled plasma spectrometry (ICP-AES, Spectro Analytical Instruments GmbH, Kleve, Germany).

### Statistical analysis

All calculations were made with STATISTICA® 12 for Windows (StatSoft Inc. 2014). The significance of differences was accepted with a 95% probability. Two-way factorial ANOVA analysis was performed with ecotypes, presence of HM in soil, and endophyte presence in plants applied as main factors. Least significant differences (LSD) were calculated according to the Fisher test. *T* tests were performed at independent samples mode for HM ion contents in leaves of *E* + and *E* − . Principal component analysis (PCA) based on the correlation matrix algorithm was performed for all chlorophyll fluorescence traits measured and calculated for all ecotypes.

## Results

### Plant collection sites

Most of the soil beneath meadows from which perennial ryegrass plants were derived, were of mineral or organic type, with medium or low soil moisture content, mainly with medium or low-intensity usage as pastures or for cutting (Supp. Table 1). All regions except one (SWK) were characterized by relatively low concentrations of HM ions in soil: Pb^2+^, c.a. 9.6; Cd^2+^, 0.17; and Cu^2+^, 4.3 [mg·kg^−1^]. Much higher (almost doubled) concentrations of HM ions have been reported by Terelak (2007) for the SWK region: Pb^2+^, c.a. 17.8; Cd^2+^, 0.37; and Cu^2+^, 7.6 [mg·kg^−1^] (Fig. 1, Supp. Table 1).

### Analysis of biomass yields, relative chlorophyll contents, and Chl a fluorescence parameters

Biomass yields were significantly affected by the ecotype and HM treatment throughout the whole experiment whereas the main effect of the endophyte was significant only for the first (after a month) and second cuts (after two months) (Table 2).

**Table 2:** Analysis of variance for the effect of ecotype, HM presence in soil and endophyte presence in plants on selected parameters of Chl *a* fluorescence (*F*_O_, *F*_M_, *F*_V_, *F*_V_/*F*_M_, *F*_V_/*F*_O_, (1-*V*j)/*V*j). *F* values were given and significance of the effects and interactions with probability higher than 99.9% (***) or 95% (**). For *T*_FM_, RC/ABS, and PI_ABS_, *F* values for none of main effects or their interactions were significant; therefore, mentioned parameters were not listed below

Source of variation	Chl a fluorescence parameters						
	*F*_O_	*F*_M_	*F*_V_	*F*_V_/*F*_M_	*F*_V_/*F*_O_	(1-*V*j)/*V*j	Area
Main effects							
Ecotype [1]	ns	ns	ns	ns	ns	ns	ns
HM in soil [2]	57.67 ^***^	31.48 ^***^	24.66 ^***^	16.70 ^***^	18.25 ^***^	9.23 ^**^	ns
Endophyte presence [3]	ns	ns	ns	ns	ns	ns	ns
Interactions							
[1] × [2]	ns	ns	ns	ns	ns	ns	ns
[1] × [3]	ns	ns	ns	ns	ns	ns	ns
[2] × [3]	27.16 ^***^	13.66 ^***^	10.36 ^**^	7.10 ^**^	9.51 ^**^	ns	15.47 ^***^
[1] × [2] × [3]	ns	ns	ns	1.90 ^**^	ns	2.19 ^**^	ns

**Fig. 2:**
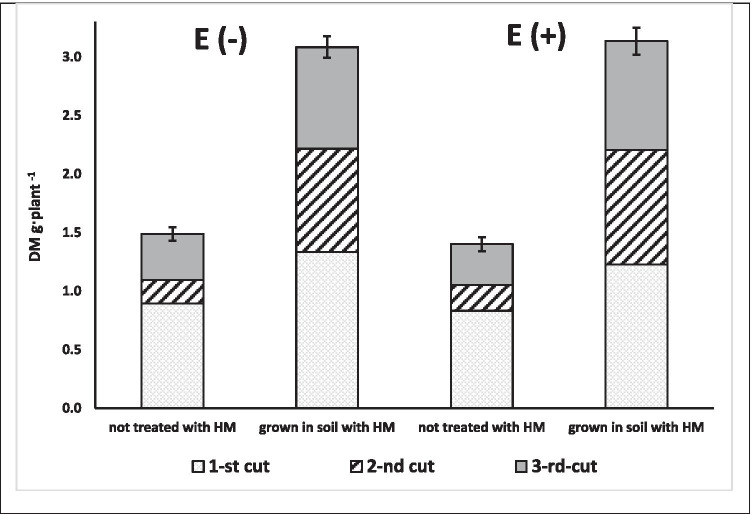
Average yields of dry biomass collected from young plants of perennial ryegrass with (*E* +) and without (*E* −) endophytes, grown in soil treated or not treated with HM. Error bar for the sum of 3 cuts

Generally, for plants grown in the presence of HM ions, dry matter yields combined for three cuts were higher (3.1 g/plants) than for control plants (1.3–1.5 g/plants) irrespective of endophyte presence in plants (Fig. 2, Supp. Figure 2). The yield of plants grown in the presence of HM, despite the presence of endophyte in plants, was 48% higher than control at 1st cut, 342% at 2nd, and 143% at 3rd cut on average. For the whole experiment, the total yield from HM treated plants was 115% higher than that of the control plants. This difference was statistically significant (*p* = 0.0000; *F* = 387.26).

**Fig. 3:**
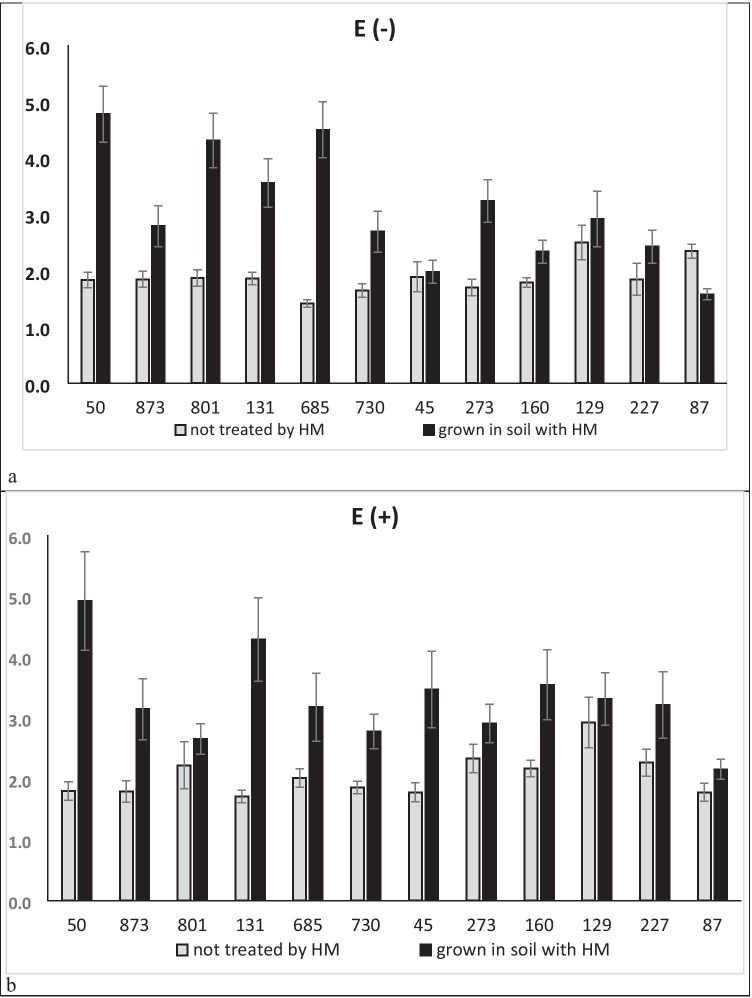
Mean values of chlorophyll contents index (CCI) in perennial ryegrass (*E −*) plants and *Epichloë*-perennial ryegrass symbiota (*E* +) grown in control conditions (left bar for each ecotype) and in the presence of HM ions (right bar for each ecotype)

Elevated concentrations of the HM in the soil, as well as the provenance of the tested ecotypes, were the main sources of variation for the relative chlorophyll content, expressed as CCI. In contrast, neither endophyte presence nor its interaction with the plant provenance and HM gave a significant effect on the CCI (Table 2). The CCI in HM treated ecotypes was on average higher than in non-HM treated ones (Fig. 3) and above difference was also significant (*p* = 0.0000; *F* = 86.21).

Elevated concentration of the HM in the soil was also the main source of variation of Chl *a* fluorescence parameters: *F*_O_, *F*_M_, F_V_, *F*_V_/*F*_M_, *F*_V_/*F*_O_, and (1-*V*j)/*V*j (Table 3, Supp. Figure 3).

**Table 3:** Analysis of variance for the effect of ecotypes, endophyte presence in the host plant and their interaction on the content of HM ions in leaves of *E* + (perennial ryegrass colonized by Epichloë endophyte) and *E* − (endophyte free perennial ryegrass). *F* values were given and significance of the effects, with probability higher than 99.9% (***)

HM ion content			
Source of variation	Pb^+2^	Cd^+2^	Cu^+2^
Ecotype [1]	124.94 ***	31.26 ***	47.87 ***
Endophyte presence [2]	ns	139.48 ***	180.79 ***
Interaction [1] × [2]	210.84***	39.22***	95.03***

**Fig. 4:**
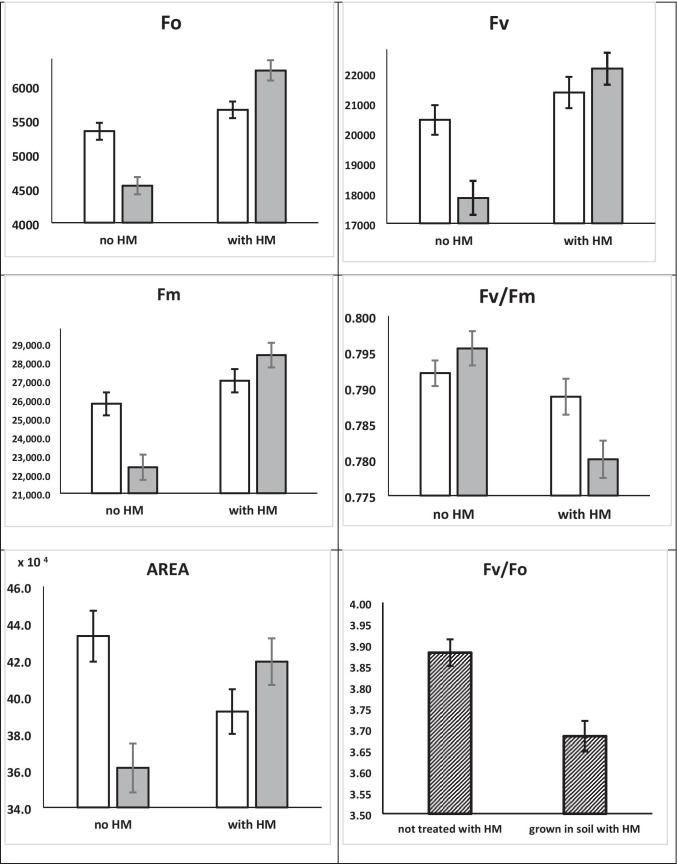
The effect of HM treatment of perennial ryegrass plants on chlorophyll *a* fluorescence parameters, which differed in statistically important manner: *F*_O_, *F*_V_, *F*_M_, Area, *F*_V_/*F*_M_, *F*_V_/*F*_O_. Bars on graph indicate standard error of means. For *F*_O_, *F*_V_, *F*_M_ arbitrary units were used

Neither the ecotype nor endophyte status resulted in a significant effect of any of the abovementioned Chl a fluorescence parameters. However, a significant interaction between HM presence in soil and endophyte presence in plants has been calculated for *F*_O_, *F*_M_, *F*_V_, *F*_V_/*F*_M_, *F*_V_/*F*_O_, and Area (Table 3, Fig. 4). For the parameters *T*_FM_, RC/ABS, and PI_ABS,_ none of the main sources of variation nor interactions were significant; therefore, they were not listed in Table 3 and Fig. 4.

Considering interactions presented in Fig. 4, perennial ryegrass plants, if grown without the addition of HM, exhibited some negative effects of endophyte presence in tissues, as reflected in lower values of *F*_M_, *F*_V_, and higher for Area. When HM was added to the soil medium, values of the mentioned parameters increased in the presence of endophytes. However, the value of the parameter reflecting the force of light reactions of PS II (*F*_V_/*F*_M_) was significantly lower in the presence of HM in soil and endophytes in plant tissues. Therefore, whether *E* + plants score higher or lower values of mentioned Chl *a* parameters than *E* − plants depends on the addition of HM to the soil medium.

Measured parameters of Chl *a* (*F*_O_, *F*_M_, *F*_V_) were influenced by HM treatment (Table 3, Supp. Figure 3). Interestingly, *E* + plants collected in more northern localities were characterized by a more visible decline of *F*_V_/*F*_M_ and *F*_V_/*F*_0_ ratios. And, as in the case of measured parameters, *E* + ecotype 730 reacted differently, by their slight increase. The ratio of *F*_V_/*F*_0_ was ≤ 4.0 in *E* − plants, whereas in *E* + plants in 3 cases, the ratio exceeded 4 (ecotypes 45, 87, and 873). Parameter (1-*V*j)/*V*j, the measure of forward electron transport, seemed to be slightly affected by HM, especially in the leaves of *E* + plants.

**Fig. 5:**
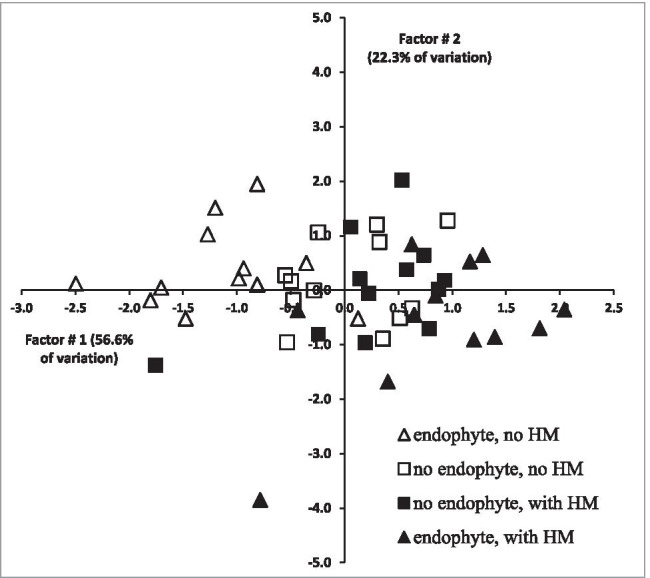
The graphical presentation of PCA analysis results based on measurements of Chl *a* parameters for ryegrass-*Epichloë* symbionts, grown with or without HM supplementation. Each data point represents a different ecotype

The PCA (principal component analysis) run on the bases of Chl *a* fluorescence parameters have shown the distribution of ecotypes depending on the endophyte presence mostly over the OX axis (first factor) (Fig. 5, Supp. Table 2) which means that most of the measured parameters, significantly correlated with the first factor (*F*_0_, *F*_V_, *F*_M_, and Area), influenced such grouping.

Ecotypes with endophytes, grown in soil without HM, were separated on the left side of the graph, as opposed to *E* + grown with the addition of HM. Negative values of factor 1, which is negatively correlated with *F*_0_, *F*_V_, *F*_M_, and Area, were ascribed to increased values of the mentioned Chl *a* parameters. On the right side of the OX axis, along with decreasing values of Chl *a* parameter, points representing *E* + plants grown with the addition of HM were located. This is another presentation of the interaction between HM and endophyte presence.

### HM ion content in E + and E − ecotypes

Analysis of variance for the data of HM ion concentration in the plant tissue revealed a statistically significant influence of both: plant provenance and endophyte presence in the host plant as well as their interaction in case of Cd^+2^ and Cu^+2^ ion concentration (Table 4).

**Table 4:** Mean values of the HM ions (Pb^+2^, Cd^+2^, Cu^+2^; mg∙kg^−1^) contents in leaves of *E* + (perennial grass colonized by *Epichloë* sp. endophyte) and *E* − (endophyte free perennial ryegrass) plants and the results of t-test for independent samples. Ecotypes were listed in decreasing order of collection sites latitude

Ecotype number	Pb^+2^			Cd^+2^			Cu^+2^		
	*E* −	*E* +	*t*	*E* −	*E* +	*t*	*E* −	*E* +	*t*
50	43.9	15.7	154.5^***^	11.8	12.3	n.s	34.7	14.6	8.0^**^
873	16.5	32.7	− 6.8^**^	8.4	8.8	n.s	10.1	37.4	− 47.1^***^
801	21.0	21.4	n.s	10.3	19.8	− 18.3^***^	15.4	15.1	n.s
131	29.3	11.0	42.1^***^	14.8	10.3	7.2^**^	23.1	13.8	9.8^**^
685	29.9	33.2	− 5.0^**^	8.0	15.1	− 34.6^***^	20.9	26.5	− 2.9^*^
730	20.8	10.2	27.5^***^	7.1	8.1	− 6.7^**^	15.1	14.6	n.s
45	32.6	23.3	12.1^***^	9.1	16.2	− 5.2^**^	25.7	30.8	− 3.7^**^
273	16.0	28.8	− 23.8^***^	11.8	10.8	3.4^*^	14.7	40.6	− 18.4^***^
160	20.2	40.7	− 15.3^***^	7.4	13.4	− 9.2^***^	16.2	47.9	− 17.5^***^
129	13.4	24.4	− 10.7^***^	10.3	13.4	− 11.2^***^	19.7	20.9	− 2.8*
227	10.4	18.9	− 14.0^***^	12.1	9.1	9.5^***^	14.0	19.6	− 6.4^**^
87	24.6	20.2	6.0^**^	10.2	12.3	n.s	21.3	22.1	n.s
Mean	23.2	23.4	n.s	10.1	12.4	− 3.44^***^	19.2	25.8	− 2.8^***^

The highest concentration of HM ions (sum of Pb^2+^, Cd^2+^, and Cu^2+^) was detected in the leaves of *E* + variant of ecotype 160 (102 mg·kg^−1^), whereas in the leaves of the *E* − plants, the concentration of HM was low (44 mg·kg^−1^) (Table 5). Differences in the particular ion concentration of the abovementioned ecotype were as follows: almost twofold higher concentration of Pb^2+^ and Cd^2+^ ions and threefold of Cu^2+^ in *E* + plants compared to *E* − .

**Table 5:** Geographical description of ecotype collection sites: decimal coordinates of northern latitude [N] and eastern longitude [E], elevation above sea level [m.a.s.l.]. The percentage share of endophyte colonized plants (Ee [%]) in each locality was shown in the last column. Ecotypes are identified by reference numbers the same across the whole manuscript

Region (code)	Ecotype number	Coordinates [decimal]		Elevation [m.a.s.l.]	Ee [%]
		Latitude [N]	Longitude [E]		
Podlaskie (POD)	50	53.653	23.138	118	57.4
Mazowieckie (MAZ)	873	52.826	21.494	100	98.1
	801	52.374	20.378	69	61.1
	131	52.195	22.482	150	87.0
	685	52.046	21.301	80	70.4
	730	51.705	21.617	128	64.8
Lubelskie (LUB)	45	50.840	21.924	155	94.4
Świętokrzyskie (SWK)	273	50.802	20.435	283	70.4
	160	50.682	20.732	284	98.1
	129	50.604	20.506	187	100.0
	227	50.539	20.935	185	64.2
	87	50.425	20.559	172	90.6

The highest concentration of Pb^2+^ (43.9 mg∙kg^−1^) was detected in the *E* − plants of ecotype 50 from POD region, whereas the lowest (10.2 mg∙kg^−1^), in the *E* + variant of ecotype 730 from MAZ region. The average value of Pb^2+^ for regions was the lowest for *E* − variants of plants from SWK region (16.9 mg∙kg^−1^), but it was also the highest (26.6 mg∙kg^−1^) for *E* + plants from the same region. Considering *E* + plants, the highest Pb^2+^ concentration (40.7 mg∙kg^−1^) was detected in ecotype 160, and was also high in ecotypes 685 and 873 (33.2 and 32.7 mg∙kg^−1^, respectively). For all those three mentioned ecotypes, Pb^2+^ concentration in *E* + plants was significantly higher than in *E* − plants. But at the same time, for other ecotypes (50, 131, 730, 45, and 87), the Pb^2+^ ion concentration was higher in *E* − plants than in *E* + .

Cadmium concentration in aerial parts of *E* + ecotypes was the highest in ecotype 801 (19.8 mg kg^−1^) as well as in ecotypes: 45 and 685 (16.2 and 15.1 mg kg^−1^, respectively) (Table 5). In a manner similar to relations described above for Pb^2+^ concentration, for all three ecotypes with relatively high Cd^2+^ concentration in *E* + plants, the Cd^2+^ ion concentration was significantly higher than the concentration values found in *E* − plants. Average concentration of Cd^+2^ in plants was similar between regions of ecotype provenance, and it ranged from 9.1 – 11.8 for *E* − plants and 11.8 to 16.2 for *E* + plants. For three from 12 tested ecotypes there were no significant difference between Cd^+2^ concentration in *E* + and *E* − plants.

High copper concentration was found in aerial parts of *E* + ecotypes 160, 273, and 873 (47.9, 40.6, and 37.4 mg·kg^−1^, respectively). All mentioned values were significantly higher than in leaves of corresponding *E* − plants. The average high concentration of Cu^2+^ ions in *E* + plants (ca. 30 mg·kg^−1^) was noted for central and southern regions, i.e., LUB and SWK. But the highest concentration of Cu^2+^ (34.7 mg·kg^−1^) was noted in *E* − plants of ecotype 50, from POD region, which was the northern most exposed.

The effect of endophyte presence in perennial ryegrass plants resulted in different types of *E* + plant reactions to elevated concentration of HM ions in the soil:

(i) *E* + plants accumulated less HM ions from the soil than *E* − plants. In the experiment there were following ecotypes: 131 (all HM ions), 50 (Pb^2+^ and Cu^2+^ ions), 730 and 87 (Pb^2+^ ions), 273 and 227 (Cd^2+^ ions);
(ii) *E* + and *E* − plants accumulated the same amounts of HM ions (no significant difference). Such was the case of ecotypes: 87 (Cd^2+^ and Cu^2+^ ions), 801 (Pb^2+^ and Cu^2+^ ions);
(iii) *E* + plants accumulated a higher amount of HM ions from soil than *E* − plants: Ecotypes 60, 129, and 685 for all HM ions; ecotypes 45, 227, 273, and 873 for two different HM ions;
(iv) variable interaction depending on HM ion: Ecotype 730 higher concentration in *E* − for Pb^2+^, higher concentration in *E* + for Cd^2+^, no difference between *E* + and *E* − for Cu^2+^.

## Discussion

There is increasing evidence that interactions of plants and microbes (including endophytes) play a critical role in metal phytoextraction and metal-mining, as they mediate different physicochemical and biological activities to facilitate ecological performances of the host plant (Muehe et al. 2015). The results of our studies revealed considerable variation in terms of the grass—fungus association's ability to cope with elevated concentration of HM ions in the soil. Mentioned ‘variation’ should be ascribed to the natural variation between host (perennial ryegrass), fungus and to their interaction. Spatial variation of mutualistic interactions between a host organism (grass plant) and infecting fungus (endophyte) through its intensity (endophyte frequency per locality) and production of toxic metabolite, i.e., ergovaline, has been previously described (Żurek et al. 2013, 2017).

Plants subjected to increased HM contents in soil were characterized by significantly higher values of the CCI and plant biomass—this could be explained based on soil fertility. The soil used for this experiment contained a low level of Cu^2+^ ions (2.4 mg·kg^−1^) and high amounts of soil organic carbon (SOC), 13%. The natural content of Cu^2+^ in soil was in the range of 15 to 40 mg·kg^−1^ in the 0 – 20 cm soil horizon and concentration of Cu^2+^ below 3.0 mg·kg^−1^ is usually defined as a deficit for grass species (Olszewska et al. 2008; Wyszkowska et al. 2013). In the presence of high organic matter content in the soil, the Cu^2+^ deficit for plants is quite frequent. Moreover, monocotyledonous plants (e.g., grasses) are particularly sensitive to Cu^2+^ deficit (Yamasaki et al. 2008). Unfortunately all these three facts together were met together in our experiment, therefore the addition of Cu to soil medium yielded better growth of HM treated plants, which was manifested in higher CCI values. The differences were not statistically significant for ecotypes collected from the southern region, except for 1 ecotype of *E* + and 2 ecotypes of *E* − which could be the result of adaptation to naturally occurring conditions of increased HM content in soil (Rodriguez et al. 2008).

Chl *a* fluorescence detection and parameters analyses (*F*_O_, *F*_M_, *F*_V_, *F*_V_/*F*_M_, *F*_V_/*F*_O_, RCB/ABS, Area, (1-*V*j)/*V*j, P_I_) are simple and widely recognized methods to assess the stress influence on plants (Żurek et al. 2014; Kalaji et al. 2016). Among fluorescence parameters measured in our experiment, *F*_O_, *F*_M_, *F*_V_, as well as the *F*_V_/*F*_M_, *F*_V_/*F*_O_ and (1-*V*j)/*V*j, were found to be significantly influenced by both HM ions addition and its interaction with endophyte status. As a reaction to stress, *F*_O_ value mostly increases, which is interpreted as lower efficiency of energy transfer between chlorophyll antennas in PS II, and our data follow reports in the literature (Prasad and Strzałka 1999). Although the increase of *F*_O_ was detected in the case of the majority of studied ecotypes, the *E* + ecotypes, compared to *E* − , were characterized by lower values of this parameter pointing to the positive influence of *Epichloë* in the host plants, as was shown in studies on host orchard grass as well (Rozpądek et al. 2015). The *F*_M_ is decreasing in response to stresses due to the fact that not all electron acceptors in PS II can be reduced. Considering results obtained in our experiment, endophyte presence in plant tissues seems to induce stress to a plant, as reflected by a decrease of *F*_M_. The *F*_V_/*F*_O_ ratio, also used for the detection of PS II destruction upon stress, can descend from values of 4–5 down to 1. According to the results obtained in this experiment, a slight but significant (< 5% on average) decrease of *F*_V_/*F*_O_ ratio was detected, showing that the stress did not influence the photosynthetic machinery to a large extent (Kalaji and Łoboda 2010).

The parameter *F*_V_/*F*_M_ is one of the most commonly used in the evaluation of plant physiological status on the bases of fluorescence characteristics. For most healthy plants, it oscillates between 0.80 and 0.83. In our experiment, it fluctuated in 0.78 and 0.81 ranges. Interestingly, HM ions induced a statistically important drop down in this parameter in *E* + ecotypes originated from northern latitudes.

The distribution of points on the PCA graph indicates that the presence of HM in soil increased stress for plants as reflected by the Chl *a* parameter describing the efficiency of PS II. Points representing the efficiency of PS II in the presence or absence of HM in soil for *E* + plants were separated over the OX axis. Considering the negative sign of correlation coefficients between factor 1, factor 2, and Chl a parameters, points on the left side of the OX axis (negative values of factor 1) represent the better status of plants than points on the right (positive values of factor 1). In the case of the absence of endophyte in host plants, there is also no clear separation of points representing the efficiency of PS II in the presence or absence of HM in soil.

Increased nutrient content due to endophyte presence was observed by many authors (Soto-Barajas et al. 2016; Malinowski et al. 2004; Zabalgogeazcoa et al. 2006). In contrast, an absence of endophyte effect for total N (Lewis et al. 1996) and Zn concentration (Monnet et al. 2005) was reported with a single perennial ryegrass genotype evaluated. In the current experiment, we have observed the whole range of possible reactions: from *E* + plants accumulating less HM than *E* − plants, through no effect, to increased accumulation of one, two, or three HM ions from the soil by *E* + plants. Detected differences resulted, probably not only from differences in the endophyte activities but also from strong interactions between the fungus and the host plant, which arose as a result of particular conditions in an origin site. In the current research, spatial aggregation of *E* + plants able to uptake relatively higher amounts of the HM from the soil has been found for Pb^2+^ accumulation. Perennial ryegrass ecotypes collected from the SWK region (locations below the latitude 50.84 N) demonstrated the ability for accumulation of relatively higher concentration of Pb^2+^ ions in *E* + plants than those from the other regions. It could be presumed that it is in line with the natural concentration of Pb^2+^ in the soils from this region which was concentrated in average of 17.8 mg·kg^−1^ of soil compared to 9.4–10.2 mg·kg^−1^ of the soils from other sampling sites in our experiment (Table S1). Hesse et al. (2003, 2004) concluded that plant-endophyte associations are adapted to their native habitats via natural selection. As we have mentioned before, the natural content of the HM, especially Pb^2+^ ions, in soil was higher in the SWK region than in other regions. Probably symbiota of this origin used to accumulate more Pb^2+^ than those coming from areas of low Pb^2+^ concentration. This could be further hypothesized that the whole microbiome of plants that came from soils of high Pb^2+^ concentration could be different from soils of low Pb^2+^ concentration. The role of the microbiome on plant health and HM tolerance has been recently widely analyzed and discussed (Dongchu et al. 2019; Ikram et al. 2018; Seneviratne et al. 2017).

The presence of HM tolerant endophytes could improve metals uptake and accumulation in hosting plants (Li et al. 2012a, b). Endophyte colonization promoted Cd^2+^ ion accumulation in tall fescue (Ren et al. 2011) and also improved Cd^2+^ transport from the root to the shoot. Hesse et al. (2003, 2004) have also found higher abundances of infected perennial ryegrass genotypes on dry sites compared to wet sites and this has been confirmed in our previous research (Żurek et al. 2013, 2017). An abundance of endophyte-infected perennial ryegrass plants was significantly and negatively correlated with annual as well as winter precipitation (multi-annual averages, 1950–2000) at localities of their origin. Considering the habitat of symbiota origin, for example, Dobrindt et al. (2013) reported higher incidences of *Neotyphodium lolii* at sites of limestone bedrock. Therefore, conditions at the place of host plant origin (both climatic and soil) may influence its ability to cope with abiotic stress (drought, soil acidity, toxic metals in soil). Differences observed between the host plants appear to depend on the endophyte and the host life histories, as well as on fungal and plant genotypes, abiotic and biotic environmental conditions, and their interactions (Saikkonen et al. 2013). Specific genotypic combinations of both host and endophyte determine the morphology and physiology of endophyte colonized grasses, as well as regulates how selective pressure acts on them (Hill et al. 1996).

## Conclusions

Tested associations (fungus + host) exerted wide variation in response to the presence of an elevated concentration of lead, cadmium, and copper in the soil. In some cases, the presence of *Epichloë* sp. in perennial ryegrass tissues resulted in the increase of accumulation of above mentioned heavy metals in aerial parts of the host plants. Generally, in the presence of endophyte mycelium, an increased accumulation of cadmium and copper was found, but not for lead.

The phyto-beneficial effect of endophytes was strongly dependent on specific host–fungus associations, which in turn could be the effect of the host plants, i.e., ecotype provenance. However, results obtained in the experiment described above are not sufficient to draw conclusions on the relationship between the provenance of symbiota and their ability to accumulate heavy metals from the soil.

To achieve the best result of the phytoremediation of heavy metals, the choice of the most effective perennial ryegrass–*Epichloë* symbiosis should be based on their laboratory evaluation.

## Supplementary Information

Below is the link to the electronic supplementary material.

Supplementary file1 (DOCX 410 KB)

## Abbreviations

ANOVA: Analysis of variance
Area: Total complementary area between the fluorescence induction curve and *F*_M_ of OJIP curve
CCl: Chlorophyll content index
Chl *a*: Chlorophyll *a*
*E*: East
*E*+: Grass-endophyte association
*E*−: Endophyte-free grass (non-colonized by endophyte)
*F*_O_: Minimal fluorescence
*F*_M_: Maximal recorded fluorescence
*F*_V_: Maximal variable fluorescence (F_M_-F_O_)
*F*_V_/*F*_M_: Maximum quantum efficiency of PSII photochemistry
*F*_V_/*F*_O_: Driving force of light reactions
(1-*V*j)/*V*j: Measure of forward electron transport
HM: Heavy metal
h: High
l: Low
LUB: Lubelskie region
m: Medium
m.a.s.l: Meters above sea level
MAZ: Mazowieckie region
min: Mineral
*n*: North
org: Organic
POD: Podlaskie region
PCA: Principal component analysis
PI_ABS_: Performance index
PS II: Photosystem II
RC/ABS: Amount of active reaction centers per absorption
SWK: ŚWiętokrzyskie region
t: T statistic
*T*_FM_: Time needed to reach the maximal fluorescence

## References

[CR1] Avila PO, Barrow JR, Lucero ME, Aaltonen RE (2012). Relationship between plant lipid bodies and fungal endophytes. Terra Latinoamericana.

[CR2] Bacon CW, Palencia ER, Hinton DM, Arora NK (2015). Abiotic and biotic plant stress-tolerant and beneficial secondary metabolites produced by endophytic bacillus species. Plant Microbes Symbiosis: Applied Facets.

[CR3] Barceló J, Poschenrieder C (1990). Plant water relations as affected by heavy metal stress: a review. J Plant Nutr.

[CR4] Bush LP, Wilkinson HH, Schardl CL (1997). Bioprotective alkaloids of grass-fungal endophyte symbioses. Plant Physiol.

[CR5] Clay K (1988). Fungal endophytes of grasses: a defensive mutualism between plants and fungi. Ecology.

[CR6] Clay K, Holah J (1999). Fungal endophyte symbiosis and plant diversity in successional fields. Science.

[CR7] Clijsters H, Van Assche F (1985). Inhibition of photosynthesis by heavy metals. Photosynth Res.

[CR8] Dobrindt L, Stroh HG, Isselstein J, Vidal S (2013). Infected–not infected: factors influencing the abundance of the endophyte Neotyphodium lolii in managed grasslands. Agr Ecosyst Environ.

[CR9] Dongchu G, Zhouzhou F, Shuyu L, Yangjiao M, Xianhong N, Fangping T, Xiawei P (2019). Changes in rhizosphere bacterial communities during remediation of heavy metal-accumulating plants around the Xikuangshan mine in southern China. Sci Rep.

[CR10] Dupont PY, Eaton CJ, Wargent JJ, Fechtner S, Solomon P, Schmid J, Day RC, Scott B, Cox MP (2015). Fungal endophyte infection of ryegrass reprograms host metabolism and alters development. New Phytol.

[CR11] Farid M, Shakoor MB, Ehsan A, Ali S, Zubair M, Hanif MS (2013). Morphological, physiological and biochemical responses of different plant species to Cd stress. Int J Chem Biochem Sci.

[CR12] Hall JL (2002). Cellular mechanisms for heavy metal detoxification and tolerance. J Exp Bot.

[CR13] Hao G, Du X, Zhao F, Ji H (2010). Fungal endophytes-induced abscisic acid is required for flavonoid accumulation in suspension cells of Ginkgo biloba. Biotech Lett.

[CR14] Hesse U, Hahn H, Andreeva K, Forster K, Warnstorff K, Schoberlein W, Diepenbrock W (2004). Investigations on the influence of Neotyphodium endophytes on plant growth and seed yield of Lolium perenne genotypes. Crop Sci.

[CR15] Hesse U, Schöberlein W, Wittenmayer L, Förster K, Warnstorff K (2003). Effects of Neotyphodium endophytes on growth, reproduction and drought-stress tolerance of three Lolium perenne L. genotypes. Grass Forage Science.

[CR16] Hill NS, Pachon JG, Bacon CW (1996). Acremonium coenophialum-mediated short- and long-term drought acclimation in tall fescue. Crop Sci.

[CR17] Hossain MA, Piyatida P, da Silva JAT, Fujita M (2012) Molecular mechanism of heavy metal toxicity and tolerance in plants: central role of glutathione in detoxification and reactive oxygen species and methylglyoxal and heavy metal chelation. J Bot 1–37

[CR18] Ikram M, Niaz A, Gul J, Farzana Gul J, Inayat Ur R, Amjad I, Hamayun M (2018). IAA producing fungal endophyte Penicillium roqueforti Thom., enhances stress tolerance and nutrients uptake in wheat plants grown on heavy metal contaminated soils. PLoS ONE.

[CR19] Kalaji HM, Jajoo A, Oukarroum A, Brestic M, Zivcak M, Samborska IA, Cetner MD, Łukasik I, Goltsev V, Ladle RJ (2016). Chlorophyll a fluorescence as a tool to monitor physiological status of plants under abiotic stress conditions. Acta Physiol Plant.

[CR20] Kalaji M, Łoboda T (2010) Chlorophyll a fluorescence in studies of the physiological state of plants. 1^st^ ed.; SGGW Warszawa, Poland, pp. 117, ISBN: 978–83–7583–119–1 (*in Polish*).

[CR21] Lewis G, Raistrick N, Bakken A, Macduff J (1996). Effect of infection by the endophytic fungus Acremonium lolii on growth and nitrogen uptake by perennial ryegrass (Lolium perenne) in flowing solution culture. Ann Appl Biol.

[CR22] Li H, Wei D, Shen M, Zhou Z (2012). Endophytes and their role in phytoremediation. Fungal Diversity.

[CR23] Li HY, Li DW, He CM, Zhou ZP, Mei T, Xu HM (2012). Diversity and heavy metal tolerance of endophytic fungi from six dominant plant species in a Pb-Zn mine wasteland in China. Fungal Ecol.

[CR24] Li X, Li W, Chu L, White JF, Xiong Z, Li H (2016). Diversity and heavy metal tolerance of endophytic fungi from Dysphania ambrosioides, a hyperaccumulator from Pb-Zn contaminated soils. J Plant Interact.

[CR25] Malinowski D, Zuo H, Belesky D, Alloush G (2004). Evidence for copper binding by extracellular root exudates of tall fescue but not perennial ryegrass infected with Neotyphodium spp. endophytes. Plant Soil.

[CR26] Monnet F, Vaillant N, Hitmi A, Sallanon H (2005). Photosynthetic activity of Lolium perenne as a function of endophyte status and zinc nutrition. Funct Plant Biol.

[CR27] Muehe E, Weigold P, Adaktylou I, Planer-Freidrich B, Kraemer U (2015). Rhizosphere microbial community composition affects cadmium and zinc uptake by the metal-hyperaccumulating plant Arabidopsis halleri. Appl Environ Microbiol.

[CR28] Nagabhyru P, Dinkins RD, Wood CL, Bacon CW, Schardl CL (2013). Tall fescue endophyte effects on tolerance to water-deficit stress. BMC Plant Biol.

[CR29] Olszewska M, Grzegorczyk S, Alberski J, Bałuch-Małecka A, Kozikowski A (2008). Effect of copper deficiency on gas exchange parameters. leaf greenness (SPAD) and yield of perrennial reygrass (Lolium perenne, L.) and ochard grass (Dactylis glomerata, L.). J Elementol.

[CR30] Paunov M, Koleva L, Vassilev A, Vangronsveld J, Goltsev V (2018). Effects of different metals on photosynthesis: cadmium and zinc affect chlorophyll fluorescence in durum wheat. Int J Mol Sci.

[CR31] Porter JK, White JF, Bacon CW (1994). Chemical constituents of grass endophytes. Biotechnology of endophytic fungi of grasses.

[CR32] Prasad MNV, Strzałka K, Springer B (1999). Impact of heavy metals on photosynthesis. heavy metal stress in plants: from molecules to ecosystems, Prasad MNV, Hagemeyer J.

[CR33] Rasmussen S, Parsons AJ, Fraser K, Xue H, Newman JA (2008). Metabolic profiles of Lolium perenne are differentially affected by nitrogen supply, carbohydrate content, and fungal endophyte infection. Plant Physiol.

[CR34] Ren A, Li C, Gao Y (2011). Endophytic fungus improves growth and metal uptake of Lolium arundinaceum Darbyshire ex Schreb. Int J Phytoremediation.

[CR35] Rodriguez RJ, Henson J, Van Volkenburgh E, Hoy M, Wright L, Beckwith F, Kim YO, Redman RS (2008). Stress tolerance in plants via habitat-adapted symbiosis. ISME J.

[CR36] Rozpądek P, Wężowicz K, Nosek M, Ważny R, Tokarz K, Lembicz M, Miszalski Z, Turnau K (2015). The fungal endophyte Epichloë typhina improves photosynthesis efficiency of its host orchard grass (Dactylis glomerata). Planta.

[CR37] Saha D, Jackson M, Johnson-Cicalese J (1988). A rapid staining method for detection of endophyte fungi in turf and forage grasses. Phytopathology.

[CR38] Saikkonen K, Gundel PE, Helander M (2013). Chemical ecology mediated by fungal endophytes in grasses. J Chem Ecol.

[CR39] Saikkonen K, Young CA, Helander M, Schardl CL (2016). Endophytic Epichloë species and their grass hosts: from evolution to applications. Plant Mol Biol.

[CR40] Schardl CL, Young CA, Faulkner JR, Florea S, Pan JL (2012). Chemotypic diversity of Epichloë, fungal symbionts of grasses. Fungal Ecol.

[CR41] Schardl CL, Young CA, Hesse U, Amyotte SG, Andreeva K, Calie PJ, Fleetwood DJ, Haws DC, Moore N, Oeser B, Panaccione DG, Schweri KK, Voisey CR, Farman ML, Jaromczyk JW, Roe BA, O'Sullivan DM, Scott B, Tudzynski P, An Z, Arnaoudova EG, Bullock CT, Charlton ND, Chen L, Cox M, Dinkins RD, Florea S, Glenn AE, Gordon A, Güldener U, Harris DR, Hollin W, Jaromczyk J, Johnson RD, Khan AK, Leistner E, Leuchtmann A, Li C, Liu J, Liu J, Liu M, Mace W, Machado C, Nagabhyru P, Pan J, Schmid J, Sugawara K, Steiner U, Takach JE, Tanaka E, Webb JS, Wilson EV, Wiseman JL, Yoshida R, Zeng Z (2013). Plant-symbiotic fungi as chemical engineers: multi-genome analysis of the *Clavicipitaceae* reveals dynamics of alkaloid loci. Plos Genetics.

[CR42] Seneviratne M, Seneviratne G, Madawala H, Vithanage M, Singh JS, Seneviratne G (2017). Role of rhizospheric microbes in heavy metal uptake by plants. Agro-Environmental Sustainability.

[CR43] Sharma SS, Dietz J (2009). The relationship between metal toxicity and cellular redox imbalance. Trends Plant Sci.

[CR44] Singh LP, Singh GS, Tuteja N (2011). Unraveling the role of fungal symbionts in plant abiotic stress tolerance. Plant Signal Behav.

[CR45] Slaughter LC, Nelson JA, Carlisle E, Bourguignon M, Dinkins RD, Philips TD, McCulley RL (2018). Climate change and Epichloë coenophiala association modify belowground fungal symbioses of tall fescue host. Fungal Ecol.

[CR46] Soleimani M, Hajabbasi MA, Afyuni M, Mirlohi A, Borggaard OK, Holm P (2010). Effect of endophytic fungi on cadmium tolerance and bioaccumulation by Festuca arundinacea and Festuca pratensis. Int J Phytorem.

[CR47] Soto-Barajas MC, Zabalgogeazcoa I, Gómez-Fuertes J, González-Blanco V, Vázquez-de-Aldana BR (2016). Epichloë endophytes affect the nutrient and fiber content of Lolium perenne regardless of plant genotype. Plant Soil.

[CR48] StatSoft Inc. (2014) STATISTICA (data analysis software system), version 12

[CR49] Strehmel N, Mönchgesang S, Herklotz S, Krüger S, Ziegler J, Scheel D (2016). Piriformospora indica stimulates root metabolism of Arabidopsis thaliana. Int J Molecul Sci.

[CR50] Tadych M, Bergen MS, White JF (2014). Epichloë spp. associated with grasses: new insights on life cycles, dissemination and evolution. Mycologia.

[CR51] Terelak H (2007) The content of trace metals and sulfur in the soils of agricultural land in Poland and soils pollution with these elements. In Vademecum of Soils Classifier, Woch, F., Ed. IUNG-PIB: Puławy, pp 224 - 240, ISBN 8389576023 (*in Polish*)

[CR52] Thijs S, Sillen W, Weyens N, Vangronsveld J (2017). Phytoremediation: state-of-the-art and a key role for the plant microbiome in future trends and research prospects. Int J Phytorem.

[CR53] Van Assche F, Clijsters H (1990). Effects of metals on enzyme activity in plants. Plant, Cell Environ.

[CR54] Wiewióra B, Żurek G, Pańka D (2015). Is the vertical transmission of Neotyphodium lolii in perennial ryegrass the only possible way to the spread of endophytes?. Plos ONE.

[CR55] Wiewióra B, Żurek G, Żurek M (2015). Endophyte-mediated disease resistance in wild population-s of perennial ryegrass (Lolium perenne L.). Fungal Ecol.

[CR56] Wyszkowska J, Borowik A, Kucharski M, Kucharski J (2013). Effect of cadmium, copper and zinc on plants, soil microorganisms and soil enzymes. J Elementol.

[CR57] Yamasaki H, Pilon M, Shikanai T (2008). How do plants respond to copper deficiency?. Plant Signal Behav.

[CR58] Zabalgogeazcoa I, Garcia Ciudad A, Vázquez de Aldana B, Garcia Criado B (2006). Effects of the infection by the fungal endophyte Epichloë festucae in the growth and nutrient content of Festuca rubra. Eur J Agron.

[CR59] Żurek G, Rybka K, Pogrzeba M, Krzyżak J, Prokopiuk K (2014). Chlorophyll a fluorescence in evaluation of the effect of heavy metal soil contamination on perennial grasses. PLoS ONE.

[CR60] Żurek G, Wiewióra B, Gozdowski D (2013). Relations between bioclimatic variables and endophytes colonization of grasses in Poland. Fungal Ecol.

[CR61] Żurek G, Wiewióra B, Żurek M, Łyszczarz R (2017). Environmental effect on Epichloë endophyte occurrence and ergovaline concentration in wild populations of forage grasses in Poland. Plant Soil.

