# Supplementary material for: Different response of perennial ryegrass—Epichloë endophyte symbiota to the elevated concentration of heavy metals in soil
Source: J Appl Genet. 2021 Sep 21;63(1):47–59. doi: 10.1007/s13353-021-00661-0 (PMC8755660; doi:10.1007/s13353-021-00661-0)
Supplement: Supplementary file 1 — Supplementary file1 (DOCX 410 KB) [file 13353_2021_661_MOESM1_ESM.docx]

Response of perennial ryegrass – *Epichloë* endophyte symbiota to the elevated concentration of heavy metals in soil

Grzegorz Żurek^1^, Barbara Wiewióra ^2,^*, Krystyna Rybka ^3^, Kamil Prokopiuk ^1^

^1^ Department of Grasses, Legumes and Energy Plants, Plant Breeding and Acclimatization Institute– National Research Institute, Radzików, Poland

^2^ Department of Seed Science and Technology, Plant Breeding and Acclimatization Institute– National Research Institute, Radzików, Poland

^3^ Department of Plant Physiology and Biochemistry, Plant Breeding and Acclimatization Institute– National Research Institute, Radzików, Poland

***** Correspondence: b.wiewiora@ihar.edu.pl

**Supplementary materials**

**Supp. Table 1.** Characteristics of collection sites: soil type (org.= organic, min.= mineral), soil moisture, average concentration of HM ions [mg∙kg^-1^] (* data according to Terelak, 2007), type and intensity of soil usage (h.= high, m= medium, l.= low).

| Region (code) | Ecotype number | Soil type | Soil moisture | HM concentration in soil [mg kg^-1^] * | | | Type and intensity of grassland usage |
| --- | --- | --- | --- | --- | --- | --- | --- |
|  |  |  |  | Cd^2+^ | Cu^2+^ | Pb^2+^ |  |
| Podlaskie (POD) | 50 | organic | high | 0.21 | 5.6 | 10.2 | mainly cut, m. |
| Mazowieckie (MAZ) | 873 | min.- org. | medium | 0.15 | 3.5 | 9.2 | mainly grazed, h. |
|  | 801 | min.- org. | medium | 0.17 | 3.7 | 9.3 | mainly grazed, m. |
|  | 131 | min.- org. | medium | 0.17 | 4.0 | 9.8 | mainly cut, h. |
|  | 685 | min.- org. | high | 0.14 | 3.7 | 9.4 | none, l. |
|  | 730 | organic | medium | 0.15 | 3.6 | 9.3 | mainly grazed, m. |
| average |  |  |  | 0.16 | 3.7 | 9.4 |  |
| Lubelskie (LUB) | 45 | mineral | low | 0.17 | 6.0 | 9.9 | none, l. |
| Świętokrzyskie (SWK) | 273 | mineral | low | 0.36 | 6.2 | 20.7 | none, l. |
|  | 160 | mineral | low | 0.36 | 6.2 | 20.7 | other, l. |
|  | 129 | organic | medium | 0.36 | 5.9 | 15.1 | mainly cut, m. |
|  | 227 | organic | medium | 0.30 | 9.8 | 15.3 | mainly cut, m. |
|  | 87 | organic | medium | 0.47 | 10.1 | 17.2 | none, l. |
| average |  |  |  | 0.37 | 7.6 | 17.8 |  |


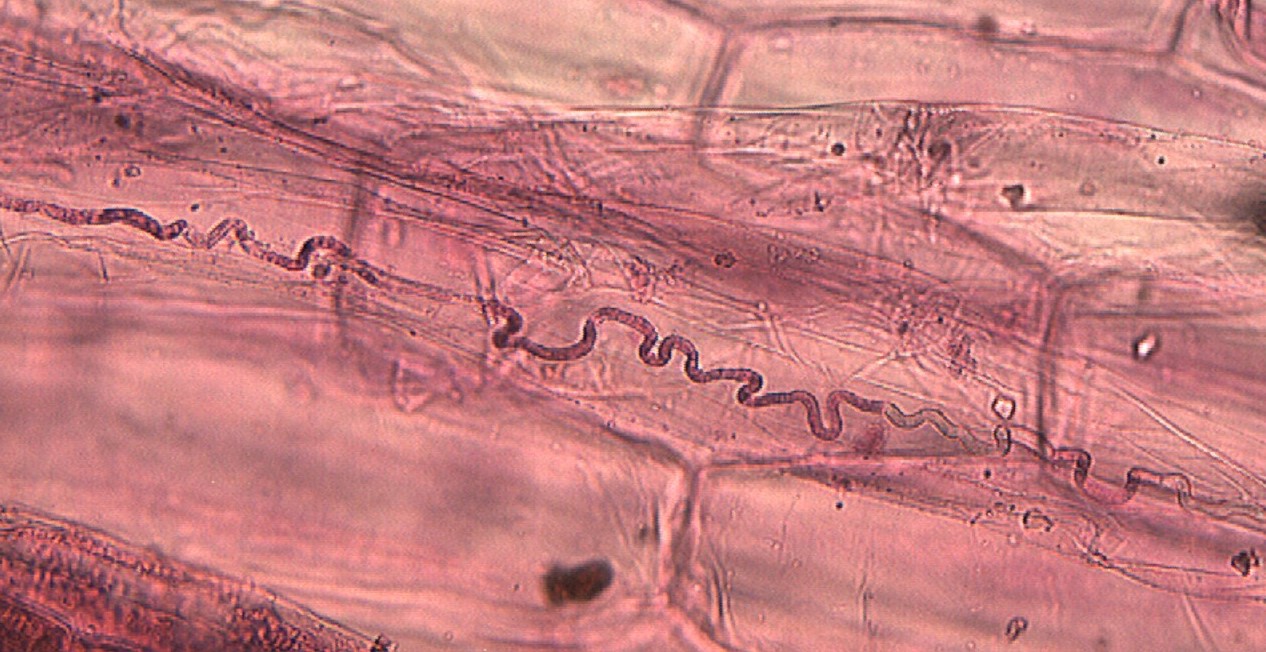


**Supp. Fig. 1.** Characteristic hyphae in the leaf intercellular spaces of perennial ryegrass.

| **1a.** |
| --- |
| **1 b.** |

**Supp. Fig. 2.** Dry mass of perennial ryegrass (E-, 1a) plants and *Epichloë*-perennial ryegrass symbiota (E+, 1b.) grown in control conditions (left bar for each ecotype number) and in the presence of HM ions (right bar for each ecotype number). Ecotypes are listed in decreasing order of collection sites latitude.

*
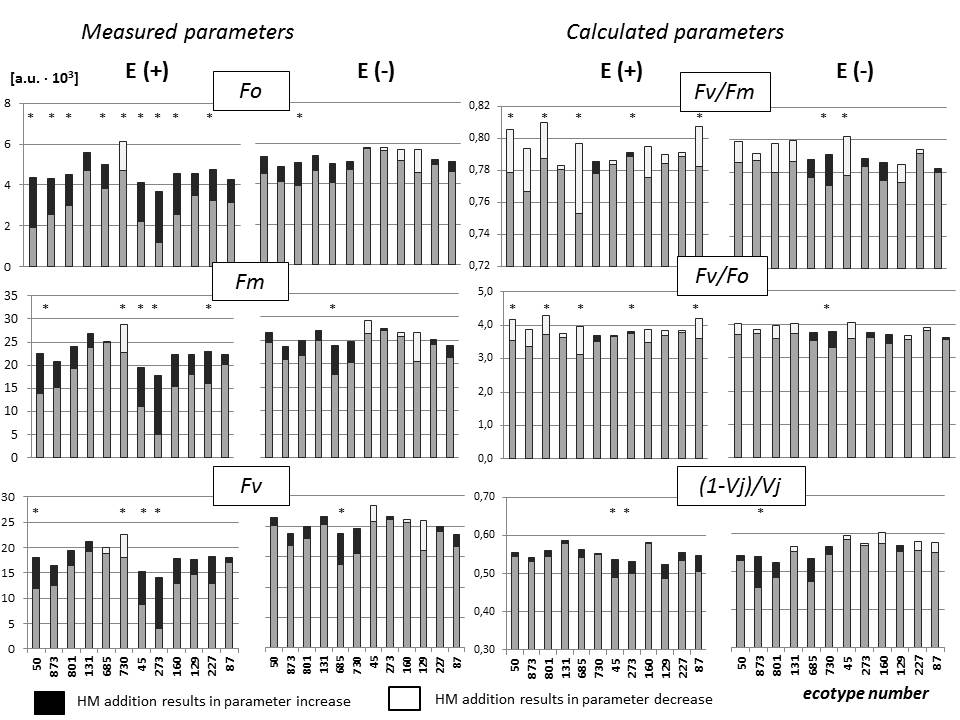
*

**Supp. Fig. 3.** Stacked column chart of changes in Chl a fluorescence parameters (measured and calculated) in response of perennial ryegrass – Epichloë symbiota (E(+) and control (E(-) to HM ions aplied to the soil. The total height of the black-grey bar corresponds to values in leaves of ecotypes treated by HM, black fragments marks increase of the parameter in response to HM as compared to control, not HM treated plants, whereas white-grey bars ilustrates the decrease of the parameter in response to HM treatment. Charts were drown for parameters found as statisticaly important on bases of Anova analysis. Ecotypes are listed in decreasing order of collection sites latitude. Intersection with a vertical axes on charts Fv/Fm and (1-Vj)/Vj is at points > 0. Significane of difference between HM treated and not treated plants was marked with asterisks (*) for α ≤ 0.05

**Supp. Table 2.** Eigenvector values of three factors calculated with Principal Component Analysis (PCA) performed on Chl *a* fluorescence parameters.

| Chl a fluorescence | Factor # | |
| --- | --- | --- |
| parameters | #1 | #2 |
| Fo | -0.983 | 0.141 |
| Fm | -0.979 | -0.148 |
| Fv | -0.951 | -0.231 |
| Fv/Fm | 0.305 | -0.716 |
| Tfm | 0.061 | 0.078 |
| Area | -0.711 | -0.279 |
| RC_ABS | -0.219 | -0.844 |
| FvFo | 0.332 | -0.729 |
| Vj | -0.460 | -0.613 |
| PI | -0.128 | -0.951 |
| variation explained (%) | 56.8 | 22.3 |

**Supp. Table 3**. Efficiency of HM extraction from soil calculated for E+ plants as a % of E-. Ecotypes were listed in decreasing order of collection sites latitude.

| **Region** | **Ecotype** | **Pb^+2^** | | **Cd^+2^** | | Cu^+2^ | | Pb^+2^ + Cd^+2^ + Cu^+2^ | |
| --- | --- | --- | --- | --- | --- | --- | --- | --- | --- |
| **(code)** | **number** | **E-** | **E+** | **E-** | E+ | E- | E+ | E- | E+ |
| POD | 50 | 100 | 35.8 | 100 | 104.2 | 100 | 42.1 | 100 | 47.1 |
| MAZ | 873 | 100 | **198.2** | 100 | 104.8 | 100 | **370.3** | 100 | **225.4** |
|  | 801 | 100 | 101.9 | 100 | **192.2** | 100 | 98.1 | 100 | 120.6 |
|  | 131 | 100 | 37.5 | 100 | 69.6 | 100 | 59.7 | 100 | 52.2 |
|  | 685 | 100 | 111.0 | 100 | **188.8** | 100 | 126.8 | 100 | 127.2 |
|  | 730 | 100 | 49.0 | 100 | 114.1 | 100 | 96.7 | 100 | 76.5 |
| LUB | 45 | 100 | 71.5 | 100 | 178.0 | 100 | 119.8 | 100 | 104.3 |
| SWK | 273 | 100 | **180.0** | 100 | 91.5 | 100 | **276.2** | 100 | **188.7** |
|  | 160 | 100 | **201.5** | 100 | **181.1** | 100 | **295.7** | 100 | **232.9** |
|  | 129 | 100 | **182.1** | 100 | 130.1 | 100 | 106.1 | 100 | 135.3 |
|  | 227 | 100 | **181.7** | 100 | 75.2 | 100 | 140.0 | 100 | 130.4 |
|  | 87 | 100 | 82.1 | 100 | 120.6 | 100 | 103.8 | 100 | 97.3 |
